# Human ACE2 Genetic Polymorphism Affecting SARS-CoV and SARS-CoV-2 Entry into Cells

**DOI:** 10.1128/spectrum.00870-22

**Published:** 2022-07-11

**Authors:** Takanari Hattori, Takeshi Saito, Kosuke Okuya, Yuji Takahashi, Hiroko Miyamoto, Masahiro Kajihara, Manabu Igarashi, Ayato Takada

**Affiliations:** a Division of Global Epidemiology, International Institute for Zoonosis Control, Hokkaido University, Sapporo, Japan; b International Collaboration Unit, International Institute for Zoonosis Control, Hokkaido University, Sapporo, Japan; c Department of Disease Control, School of Veterinary Medicine, University of Zambia, Lusaka, Zambia; Pontificia Universidad Católica de Chile

**Keywords:** ACE2, polymorphism, SNPs, SNVs, SARS-CoV-2, SARS-CoV, spike protein, viral entry

## Abstract

Severe acute respiratory syndrome coronavirus (SARS-CoV) and SARS-CoV-2 have a single envelope glycoprotein (S protein) that binds to human angiotensin-converting enzyme 2 (ACE2) on the host cell membrane. Previous mutational scanning studies have suggested that some substitutions corresponding to single nucleotide variants (SNVs) in human ACE2 affect the binding affinity to the receptor binding domain (RBD) of the SARS-CoV-2 S protein. However, the importance of these substitutions in actual virus infection is still unclear. In this study, we investigated the effects of the reported ACE2 SNV substitutions on the entry of SARS-CoV and SARS-CoV-2 into cells, using vesicular stomatitis Indiana virus (VSIV) pseudotyped with S proteins of these coronaviruses (CoVs). HEK293T cells transfected with plasmids expressing ACE2 having each SNV substitution were infected with the pseudotyped VSIVs and relative infectivities were determined compared to the cells expressing wild-type ACE2. We found that some of the SNV substitutions positively or negatively affected the infectivities of the pseudotyped viruses. Particularly, the H505R substitution significantly enhanced the infection with the pseudotyped VSIVs, including those having the substitutions found in the S protein RBD of SARS-CoV-2 variants of concern. Our findings suggest that human ACE2 SNVs may potentially affect cell susceptibilities to SARS-CoV and SARS-CoV-2.

**IMPORTANCE** SARS-CoV and SARS-CoV-2 are known to cause severe pneumonia in humans. The S protein of these CoVs binds to the ACE2 molecule on the plasma membrane and mediates virus entry into cells. The interaction between the S protein and ACE2 is thought to be important for host susceptibility to these CoVs. Although previous studies suggested that some SNV substitutions in ACE2 might affect the binding to the S protein, it remains elusive whether these SNV substitutions actually alter the efficiency of the entry of SARS CoVs into cells. We analyzed the impact of the ACE2 SNVs on the cellular entry of SARS CoVs using pseudotyped VSIVs having the S protein on the viral surface. We found that some of the SNV substitutions positively or negatively affected the infectivities of the viruses. Our data support the notion that genetic polymorphisms of ACE2 may potentially influence cell susceptibilities to SARS CoVs.

## INTRODUCTION

Coronaviruses (CoVs), which belong to the family *Coronaviridae* in the order *Nidovirales*, are enveloped positive-sense single-stranded RNA viruses. The genus *Betacoronavirus* is one of the four genera in the subfamily *Orthocoronavirinae* and includes severe acute respiratory syndrome coronavirus (SARS-CoV), SARS-CoV-2, and Middle East respiratory syndrome coronavirus, all of which are known to cause severe pneumonia in humans. Since its first report in China, coronavirus disease 2019 (COVID-19) caused by SARS-CoV-2 has spread all over the world. As of 2 March 2022, there have been 437,333,859 confirmed cases of COVID19, including 5,960,972 deaths, as indicated by the World Health Organization (WHO) COVID-19 report (https://covid19.who.int/), accessed on 3 March 2022.

The spike (S) protein of SARS-CoV and SARS-CoV-2 is a single envelope glycoprotein that is responsible for virus entry into cells and thought to be important for host range restriction of these CoVs (1, 2). The S protein is the primary determinant of antigenicity and thus the only target of neutralizing antibodies (1, 2). The mature S protein consists of two subunits, S1 and S2, which are cleaved by host proteases during the post translational processing (3). The S1 subunit contains the receptor binding domain (RBD), which recognizes angiotensin-converting enzyme 2 (ACE2) as a receptor, leading to viral attachment to the host cell (3). Subsequently, the S2 subunit is further cleaved at the S1/S2 and S2′ sites by host proteases such as furin, transmembrane protease serine 2 (TMPRSS2), and cathepsins on the surface and in the endosomes of target cells. After the cleavage of the S protein, the S2 subunit induces membrane fusion between the viral envelope and host cell membranes (4, 5).

During the current SARS-CoV-2 pandemic, some particular SARS-CoV-2 lineages are classified as variants of concern (VOCs), such as the Alpha (lineage B.1.1.7), Beta (lineage B.1.351), Gamma (lineage P1), and Delta (lineage B.1.617.2) variants, which are thought to be associated with increased transmissibility and infectivity (6). Focusing on the mutations in the RBD of these VOCs, the N501Y mutation found in the Alpha variant, K417N, E484K, and N501Y mutations found in the Beta variant, K417T, E484K, and N501Y mutations found in the Gamma variant, and L452R and T478K mutations found in the Delta variant are reported to enhance the binding affinity of the S protein to the ACE2 receptor and also to be important for escape from neutralization by several monoclonal antibodies (7).

The severity of COVID-19 symptoms differs among individuals, ranging from asymptomatic or only mild cold-like symptoms to pneumonia with a severe clinical course (8). While the pathogenesis of COVID-19 is thought to be related to viral tropism, host cell-mediated immunity, and the inflammatory response (9), host genetic polymorphisms in humans have also been suggested to be one of the factors determining the disease severity of SARS-CoV-2 infection (10). Since ACE2 is the key functional host receptor for SARS-CoV-2, genetic diversity in this receptor may potentially be involved in the difference of SARS-CoV-2 infectivity among individuals. ACE2 is expressed in various tissues, including lung, kidney, intestine, and blood vessels (11) and plays an important role in controlling blood pressure by regulating the renin-angiotensin-aldosterone system (12). Several studies have suggested that single nucleotide variants (SNVs), including single nucleotide polymorphisms (SNPs), in ACE2 could potentially change the efficiency of SARS-CoV-2 infection by affecting the affinity of ACE2 to the SARS-CoV-2 S protein or the cell surface expression of ACE2 (13–15). Another group examined the impact of eight ACE2 SNVs found in specific populations focusing on the cellular entry of SARS-CoV-2 and suggested that these SNV substitutions had limited impact on the efficiency of ACE2-mediated entry of SARS-CoV-2 (16). However, many ACE2 SNVs remain to be investigated to determine their importance for SARS-CoV-2 infection.

Using a deep mutational scanning method, a previous study showed the effects of 117 single amino acid substitutions of ACE2, all located on the interface with the SARS-CoV-2 S protein, which are present in the angiotensin peptide-binding cavity (17). Of these, 31 ACE2 SNV substitutions were found to affect the binding affinity of ACE2 to the RBD of the SARS-CoV-2 S protein (17, 18). However, it is still unknown whether these SNV substitutions affect cell susceptibilities to the virus. In this study, we virologically analyzed the impact of the ACE2 SNVs on the cellular entry of SARS-CoV and SARS-CoV-2 using vesicular stomatitis Indiana viruses (VSIVs) pseudotyped with the S protein. Four SARS-CoV-2 VOCs were also investigated. Our data suggest that ACE2 SNVs may potentially influence host susceptibilities to SARS-CoV and SARS-CoV-2, including VOCs.

## RESULTS

### Selection of ACE2 SNVs for the analysis.

**FIG 1:**
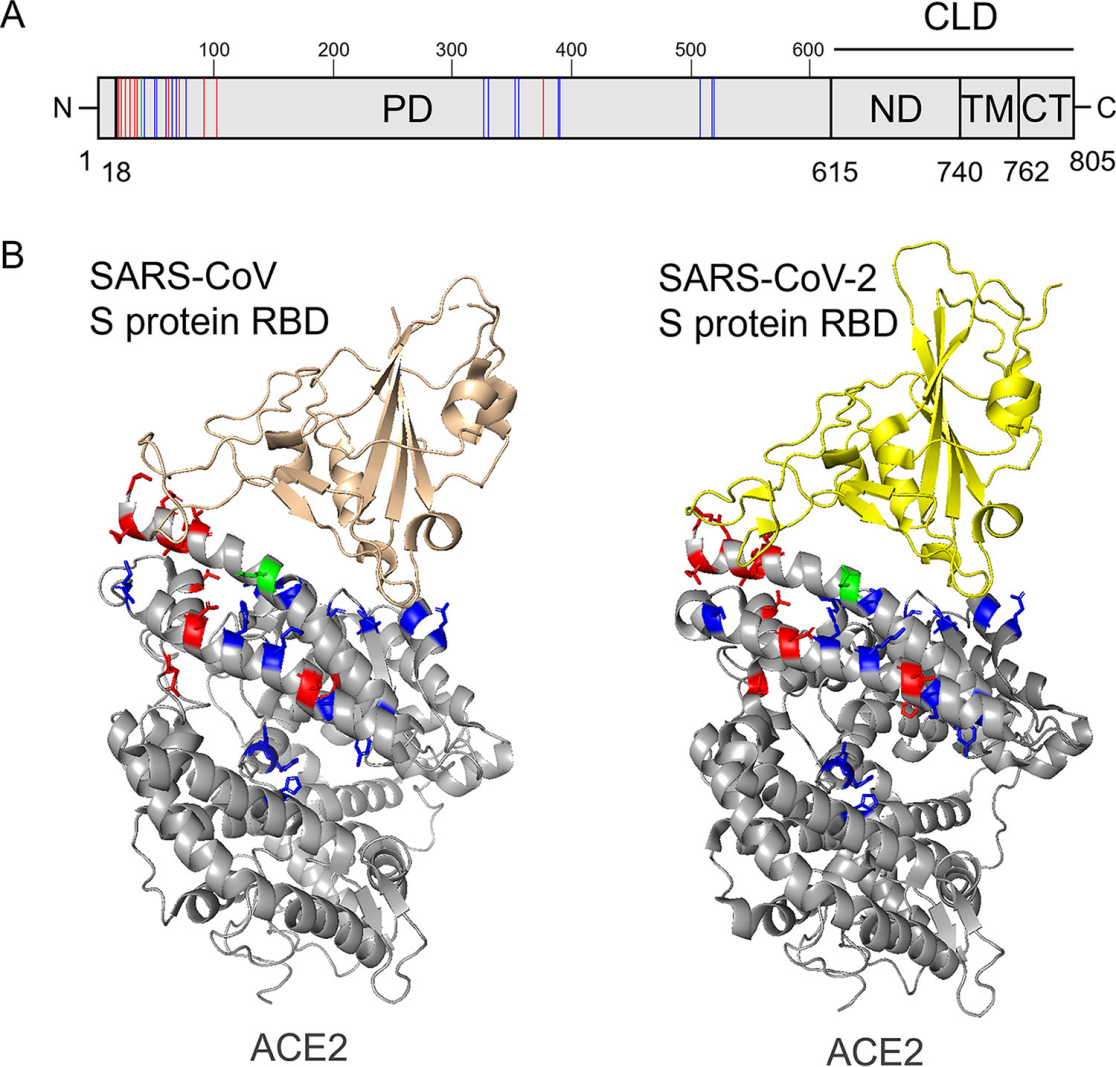
Structures of ACE2 and SARS CoV S proteins. (A) Schematic diagram of human ACE2. ACE2 is a type I membrane protein, consisting of an N-terminal signal peptide, an extracellular domain containing a protease domain (PD) and neck domain (ND), a transmembrane domain (TM), and a cytoplasmatic tail (CT). ND, TM, and CT form the collectrin-like domain (CLD). The amino acid positions of ACE2 SNVs that were reported to increase and decrease the interaction to the RBD of the SARS-CoV-2 S protein are depicted as red and blue lines, respectively. The substitution at position 35 (E35D and E35K) of ACE2 showing inconsistent effects is depicted as a green line. (B) Crystal structures of the complex of ACE2 and SARS CoV S proteins. The structural data were obtained from Protein Data Bank (PDB; https://www.rcsb.org/; PDB ID 2AJF and 6lzg). Human ACE2, SARS-CoV RBD, and SARS-CoV-2 RBD polypeptides are shown as ribbon models in gray, pink, and yellow, respectively. Amino acid positions of ACE2 SNVs that were reported to increase and decrease the interaction to the RBD of the SARS-CoV-2 S protein are shown in red and blue, respectively. The substitution at position 35 (E35D and E35K) of ACE2 showing inconsistent effects is shown in green. The amino acid positions indicated in panel A are shown in the same colors.

Using deep mutational scanning analyses of ACE2, amino acid substitutions that altered the binding affinity to RBD of the SARS-CoV-2 S protein were previously identified (17). Of these, 31 substitutions coincided with the reported nonsynonymous SNV substitutions in ACE2 (18). These studies showed that 13 SNV substitutions (S19P, I21V, I21T, E23K, A25T, K26R, T27A, E35D, N64K, E75G, T92I, Q102P, and H378R) increased the interaction with the RBD of the SARS-CoV-2 S protein, whereas 18 SNV substitutions (E35K, E37K, Y50F, N51D, N51S, M62V, K68E, F72V, M82I, G326E, E329G, G352V, D355N, Q388L, P389H, H505R, R514G, and Y515C) showed negative effects for the ACE2-RBD binding. Since the I21V/T and N51D/S mutations were shown to have similar effects on the RBD-ACE2 interaction, we selected I21T and N51D for further analyses. In total, 29 nonsynonymous SNVs were analyzed for the following experiments (Table 1). The ACE2 molecule (805 amino acids) consists of the protease domain (PD), which is known to interact with RBD of the S protein, and the collectrin-like domain (CLD), which contains the neck domain (ND), transmembrane domain (TM), and cytoplasmic tail (CT) (Fig. 1A). All 29 SNV positions were found in PD (Fig. 1B).

**TABLE 1:** Information on human ACE2 SNV mutants validated in this study

rsID^*a*^	Position	Amino acids (wild type/mutant)	SNV	Binding affinity to RBD of SARS-CoV-2 S^*b*^
rs73635825	19	S/P	S19P	Increased
rs1244687367	21	I/T	I21T	Increased
rs756231991	23	E/K	E23K	Increased
rs1434130600	25	A/T	A25T	Increased
rs4646116	26	K/R	K26R	Increased
rs781255386	27	T/A	T27A	Increased
rs778500138	35	E/D	E35D	Increased
rs1348114695	35	E/K	E35K	Decreased
rs146676783	37	E/K	E37K	Decreased
rs1192192618	50	Y/F	Y50F	Decreased
rs760159085	51	N/D	N51D	Decreased
rs1325542104	62	M/V	M62V	Decreased
rs1199100713	64	N/K	N64K	Increased
rs755691167	68	K/E	K68E	Decreased
rs1256007252	72	F/V	F72V	Decreased
rs867318181	75	E/G	E75G	Increased
rs766996587	82	M/I	M82I	Decreased
rs763395248	92	T/I	T92I	Increased
rs1395878099	102	Q/P	Q102P	Increased
rs759579097	326	G/E	G326E	Decreased
rs143936283	329	E/G	E329G	Decreased
rs370610075	352	G/V	G352V	Decreased
rs961360700	355	D/N	D355N	Decreased
rs142984500	378	H/R	H378R	Increased
rs751572714	388	Q/L	Q388L	Decreased
rs762890235	389	P/H	P389H	Decreased
rs1016409802	505	H/R	H505R	Decreased
rs1352194082	514	R/G	R514G	Decreased
rs1263424292	515	Y/C	Y515C	Decreased

a NCBI ID numbers of ACE2 SNVs.

b The effects of the substitutions on the binding affinity to the RBD of the SARS-CoV-2 S protein were obtained from previous studies (17, 18).

### ACE2 SNV substitutions that affected the cellular entry of SARS-CoV and SARS-CoV-2.

**FIG 2:**
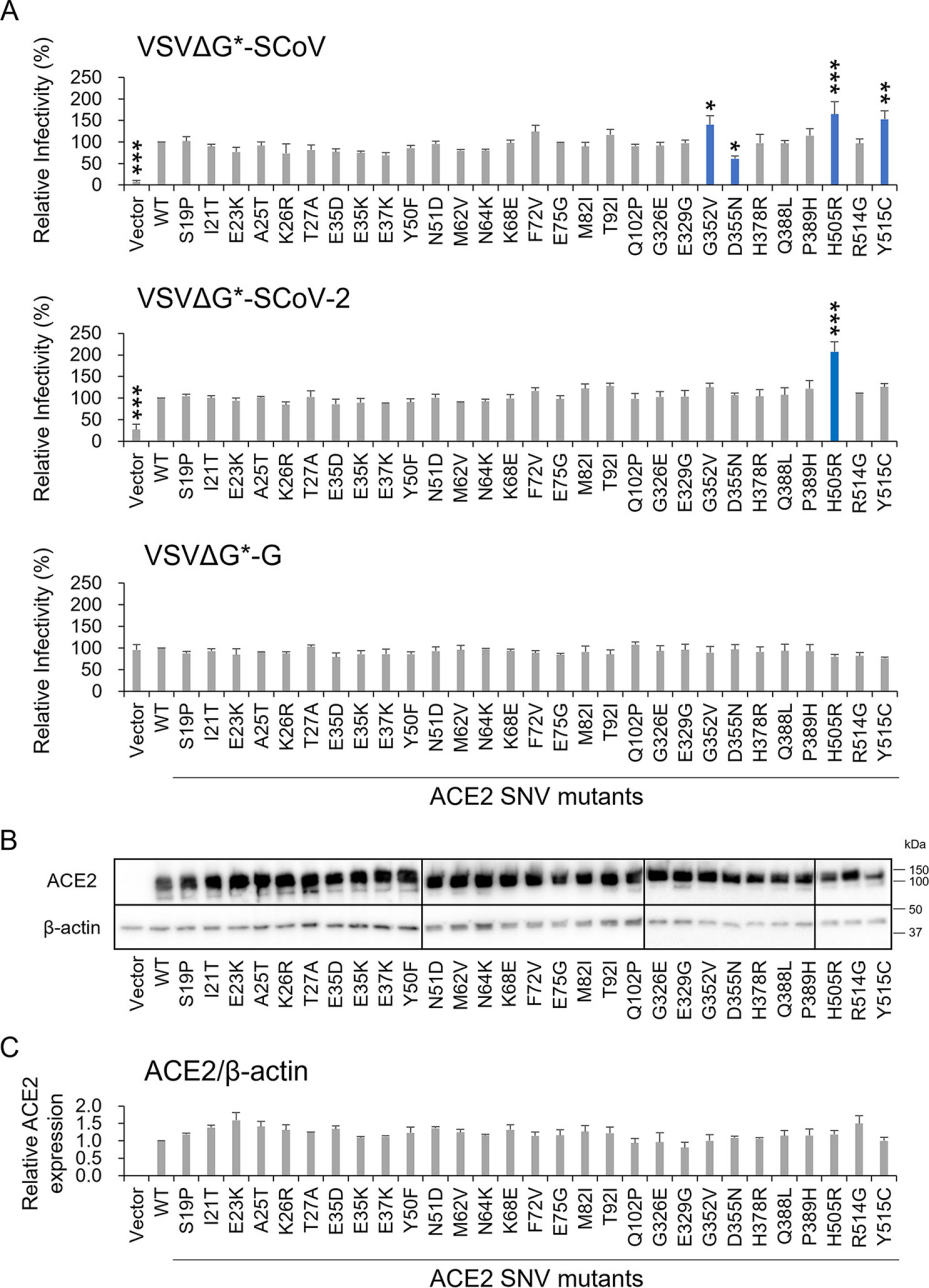
Effects of ACE2 SNV substitutions on the infectivity of VSIVs pseudotyped with the S protein of SARS-CoV and SARS-CoV-2. (A) HEK293T cells transfected with the plasmids expressing WT ACE2 or its SNV mutants were infected with VSVΔG*-SCoV, VSVΔG*-SCoV-2, and VSVΔG*-G. IUs were determined by counting the numbers of GFP-expressing cells at 24 h postinfection. Relative infectivities of pseudotyped VSIVs in the cells transfected with each plasmid were determined by setting the IU value given by HEK293T cells expressing exogenous WT ACE2 to 100%. The means and standard deviations of three independent experiments are shown. Statistical significance was calculated compared to the WT using the Dunnett test (*, *P < *0.05; **, *P < *0.01; ***, *P < *0.001). (B) HEK293T cells transfected with the plasmids expressing WT ACE2 or its SNV mutants were harvested at 48 h posttransfection and analyzed by SDS-PAGE and Western blotting. The amounts of β-actin in the total cell lysate were also analyzed as an internal control. (C) The band intensities of WT and mutant ACE2 molecules were compared. The ratios of the expression levels of ACE2 were analyzed. The value of WT ACE2 was set to 1.0. The means and standard deviations of three independent experiments are shown. Statistical significance was calculated using the Dunnett test and no significant difference was detected between WT and mutant proteins.

**FIG 3:**
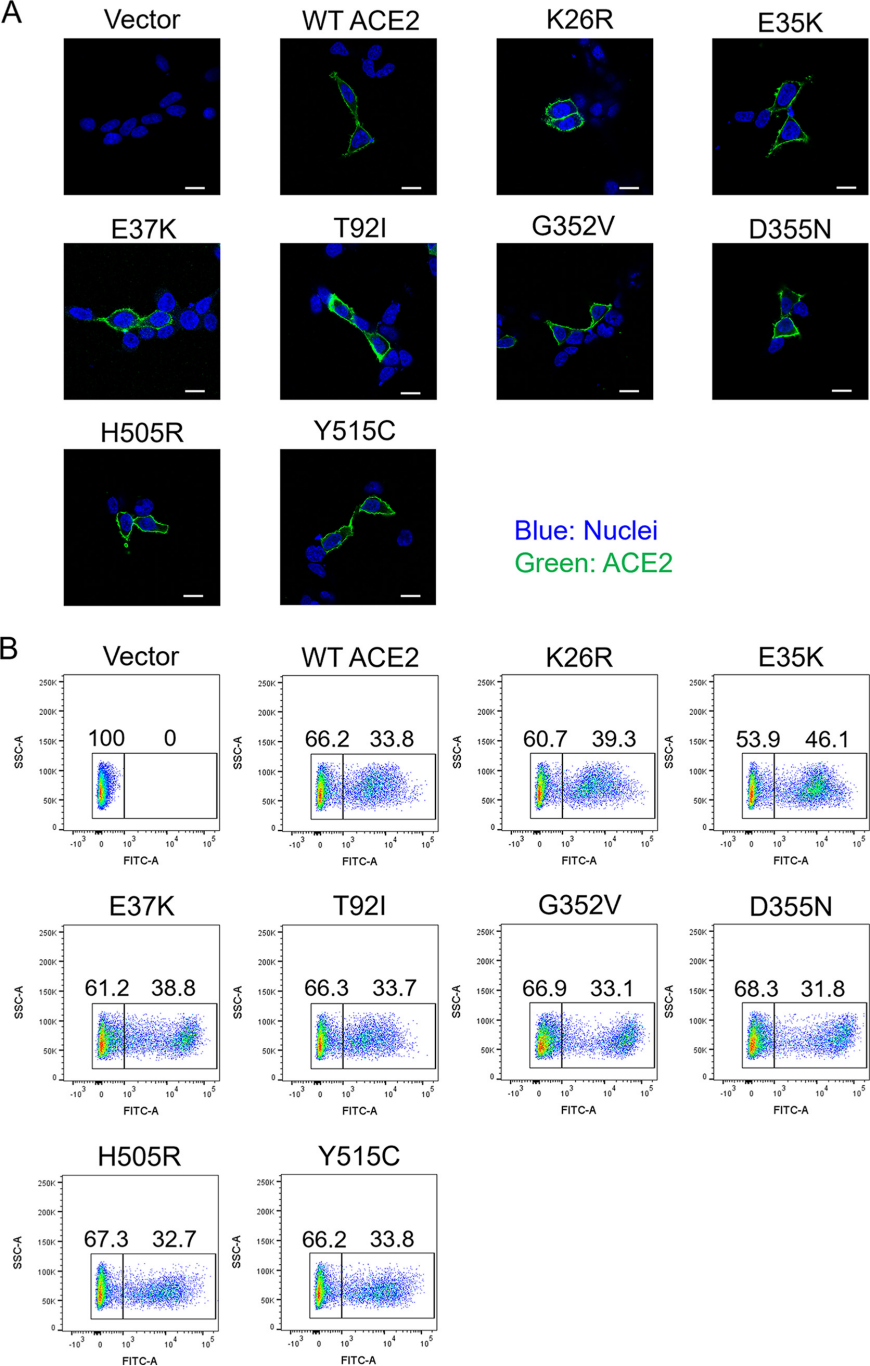
Cell surface expression of ACE2 in HEK293T cells. (A) HEK293T cells transfected with the ACE2-expressing plasmids were immunostained with an anti-ACE2 monoclonal antibody and Alexa Fluor 488-conjugated goat anti-rabbit IgG and analyzed by confocal microscopy. Nuclei of cells were stained with DAPI. Scale bars, 10 μm. (B) The surface expression of exogenously introduced ACE2 was analyzed by flow cytometry. HEK293T cells transfected with the plasmids were immunostained with the same antibodies as described above. After washing, the cells were analyzed using a FACSCanto flow cytometer.

To evaluate the influence of these ACE2 SNV substitutions on the cellular entry of SARS CoVs, HEK293T cells expressing exogenous ACE2 wild-type (WT) and SNV mutants were infected with VSIVs pseudotyped with the S proteins of SARS-CoV (VSVΔG*-SCoV), SARS-CoV-2 (VSVΔG*-SCoV-2), and VSIV G protein (VSVΔG*-G), and the relative infectivities were compared to those of the cells expressing WT ACE2 (Fig. 2A). Although we observed SARS-CoV and -CoV-2 entry into empty vector-transfected cells at low titers, the infectious units (IUs) were increased significantly by the expression of exogenous ACE2, suggesting that the entry via the alternative pathways (19) had limited impact in the comparison among the ACE2 proteins in our experimental conditions. We found that some of the ACE2 SNV substitutions positively or negatively affected the infectivity of VSVΔG*-SCoV and/or VSVΔG*-SCoV-2. G352V and Y515C substitutions in ACE2 significantly enhanced the entry of VSVΔG*-SCoV but not VSVΔG*-SCoV-2. H505R substitution significantly enhanced the entry of both VSVΔG*-SCoV and VSVΔG*-SCoV-2. On the other hand, the D355N substitution significantly reduced the infectivity of VSVΔG*-SCoV but not VSVΔG*-SCoV-2. These results indicated that the amino acid residue at position 505 of ACE2 was commonly important for cell susceptibilities to both SARS-CoV and SARS-CoV-2, whereas the roles of the amino acid residues at positions 352, 355, and 515 in the interaction with the S protein might be different between SARS-CoV and SARS-CoV-2. As expected, none of the examined SNV substitutions significantly affected the infectivity of VSVΔG*-G. We confirmed that most of the ACE2 SNV mutants exogenously introduced into HEK293T cells were expressed to similar extents, except for a few mutants such as E23K and E329G that did not affect the viral infectivity (Fig. 2B and C). Using immunofluorescence assay and flow cytometric analysis, we confirmed that these representative ACE2 SNV mutants (G352V, D355N, H505R, and Y515C) were similarly localized on the cell surface, as was the case with WT ACE2 (Fig. 3). Taken together, these results suggested that SNV substitutions of human ACE2 affected the entry of SARS CoVs into cells.

### ACE2 SNV substitutions that affected the cellular entry of SARS-CoV-2 VOCs.

**FIG 4:**
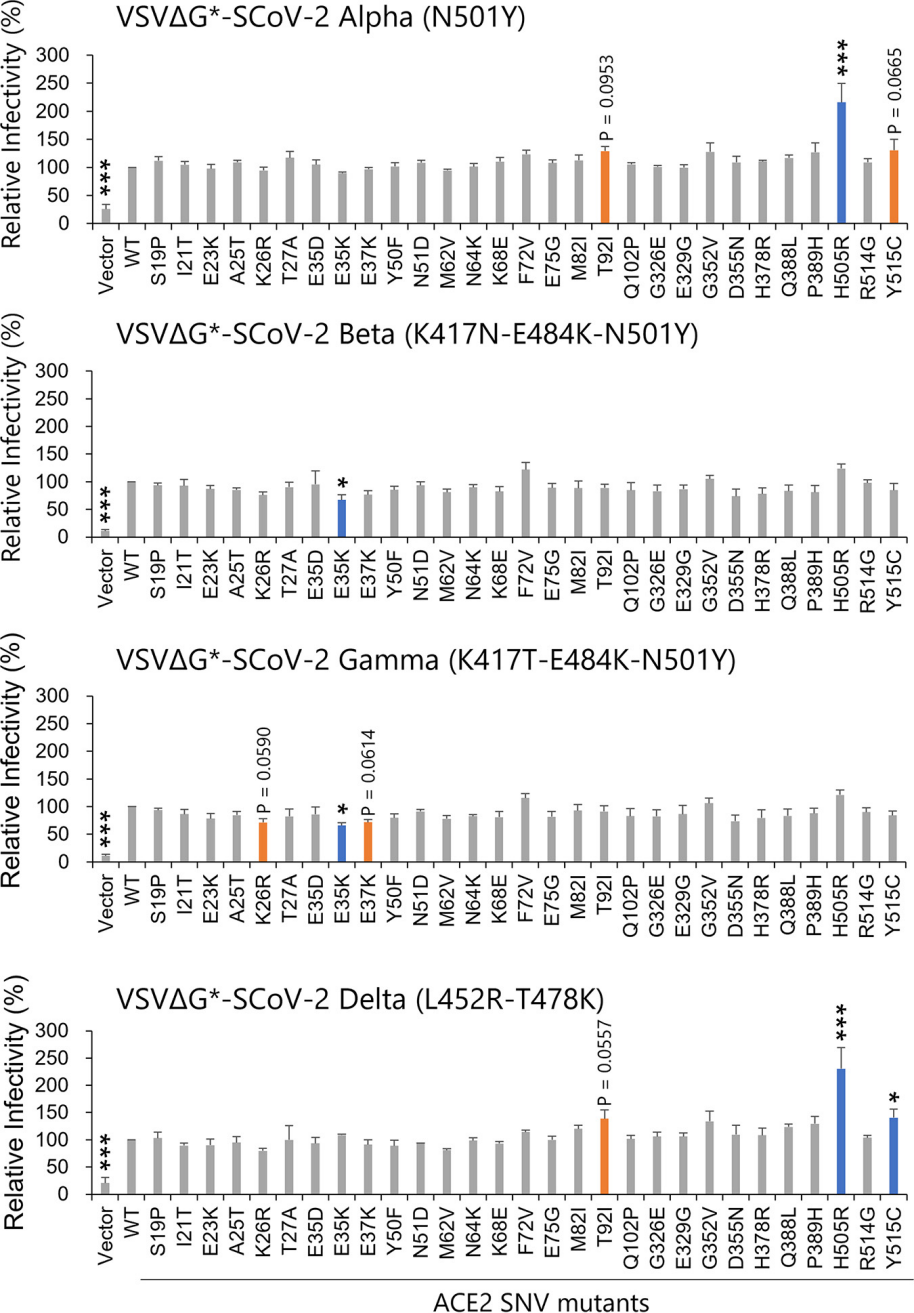
Effects of ACE2 SNV substitutions on the infectivity of VSIVs pseudotyped with the S protein with VOC mutations. HEK293T cells transfected with the plasmids expressing WT ACE2 or its SNV mutants were infected with VSIVs pseudotyped with S proteins having RBD substitutions found in SARS-CoV-2 Alpha (N501Y), Beta (K417N, E484K, and N501Y), Gamma (K417T, E484K, and N501Y), and Delta (L452R and T478K) variants. The relative infectivities of pseudotyped VSIVs were determined as described in the legend of Fig. 1. The means and standard deviations of three independent experiments are shown. Statistical significance was calculated compared to the WT using the Dunnett test (*, *P < *0.05; **, *P < *0.01; ***, *P < *0.001).

We next investigated whether the 29 SNVs could affect the cellular entry of VOCs (Alpha, Beta, Gamma, and Delta) of SARS-CoV-2, which had some signature substitutions in RBD of the S protein (Fig. 4). We prepared VSIVs pseudotyped with the S protein having VOC-derived substitutions in RBD of the S protein and determined infectivities in HEK293T cells expressing each ACE2 protein. Infectivities of VSVΔG*-SCoV-2 Alpha (N501Y) and Delta (L452R-T478K) were significantly enhanced by the H505R substitution (*P = *0.001). The Y515C substitution also promoted the infection with SCoV-2 Delta (*P = *0.0383). In contrast, these substitutions did not significantly affect the infectivity of VSVΔG*-SCoV-2 Beta (K417N-E484K-N501Y) or Gamma (K417T-E484K-N501Y). Interestingly, however, the infectivities of VSVΔG*-SCoV-2 Beta and Gamma variants were significantly reduced by the E35K substitution (*P = *0.0266 and 0.0125, respectively) but those of VSVΔG*-SCoV-2 Alpha and Delta variants were not. Although not statistically significant compared to HEK293T expressing WT ACE2, some other ACE2 substitutions slightly affected the infection: T92I and Y515C enhanced VSVΔG*-SCoV-2 Alpha (*P = *0.0953 and 0.0665, respectively), K26R and E37K reduced VSVΔG*-SCoV-2 Beta (*P = *0.059 and 0.061), and T92I enhanced VSVΔG*-SCoV-2 Delta (*P = *0.0557). We confirmed that these ACE2 SNV mutants (K26R, E35K, E37K, and T92I) were also localized on the cell surface (Fig. 3). These data suggested that effects of ACE2 SNV substitutions on SARS-CoV-2 entry into cells might be different among the SARS-CoV-2 variants.

### Molecular position and allele frequencies of the selected ACE2 SNV mutants.

**FIG 5:**
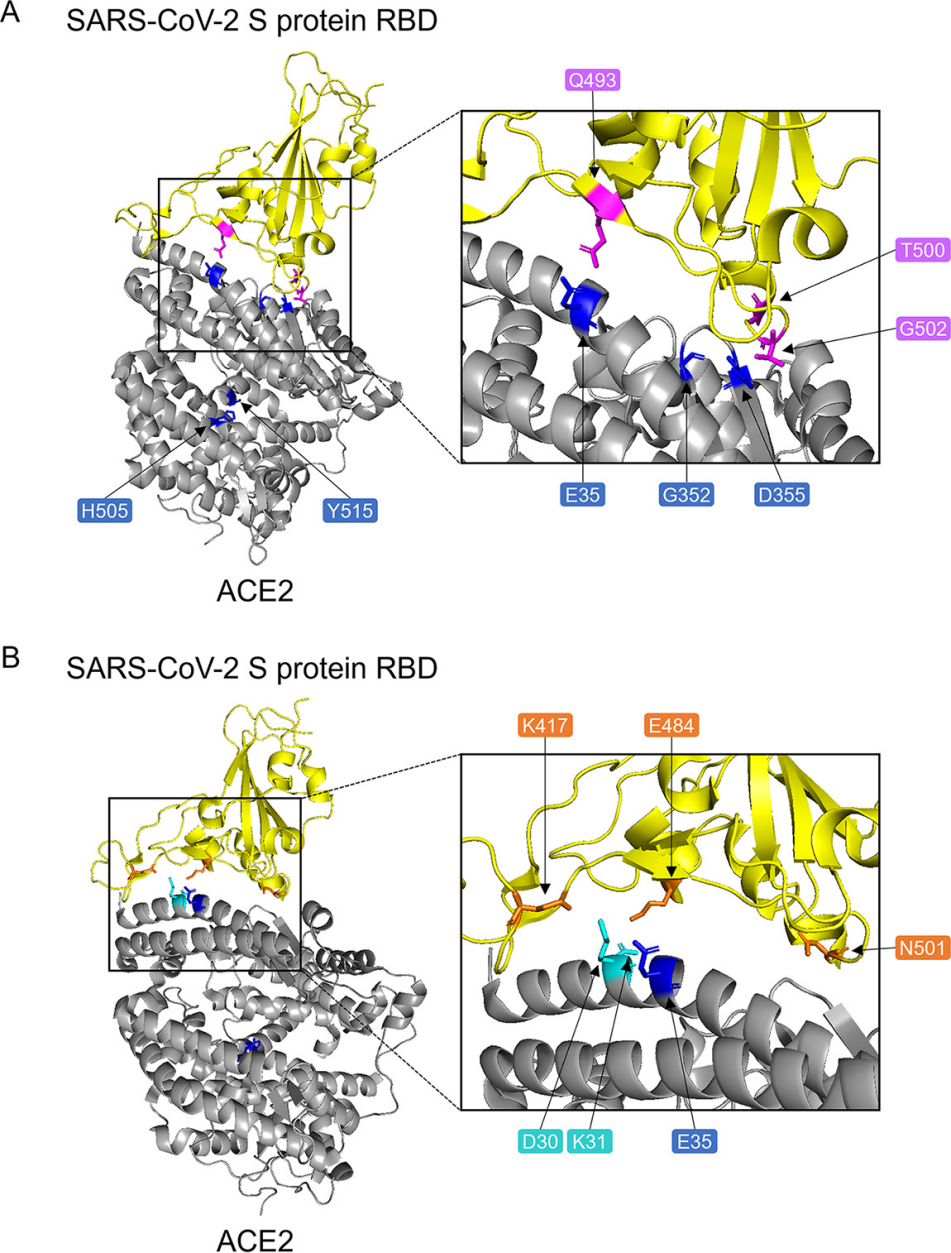
Amino acid positions of the ACE2 SNV substitutions that affected the cellular entry of SARS-CoV and SARS-CoV-2. The crystal structure of the complex of ACE2 and the SARS-CoV-2 S protein (PDB ID 6lzg). The amino acid positions of five ACE2 SNVs that affected the cellular entry of SARS-CoV and/or SARS-CoV-2 are shown in blue. (A) Two amino acid residues (T500 and G502) and Q493 in the S protein of SARS-CoV-2, which are shown in magenta, are known to bind to D355 and E35 of ACE2, respectively. (B) Three amino acid positions (K417, E484, and N501) at which substitutions were found in the S protein of SARS-CoV-2 Beta and Gamma variants are shown in orange. K417 and E484 in the S protein, which are known to bind to D30 and K31 of ACE2, respectively, are shown in cyan.

We found that five SNV substitutions in ACE2 (E35K, G352V, D355N, H505R, and Y515C) significantly affected the cellular entry of SARS CoVs. E35K, G352V, and D355N were involved in the interaction site with the S protein, whereas H505R and Y515C were located far from the interaction site (Fig. 5A). Using public SNP databases, Trans-Omics for Precision Medicine (TOPMed), Genome Aggregation Database (genomAD), and Exome Aggregation Consortium (ExAC), we compared the allele frequencies of these mutants (Table 2). E35K, G352V, D355N, H505R, and Y515C are uncommon in the global population (minor allele frequency [MAF] = 0.00001, 0.00003, 0.00001, 0.00001, and 0.000004, respectively). E35K is more frequently found in the East Asian population (MAF = 0.0001) compared to the global population. Thus, the allele frequencies of the SNV mutants that could potentially alter the cell susceptibility to SARS CoVs was found to be very low in the current population.

**TABLE 2:** Minor allele frequencies of human ACE2 SNV substitutions that affected the entry of SARS CoVs

SNV	Minor allele frequency^*a*^					
	Global	African	American	East Asian	South Asian	European
E35K	0.00001	0	0	0.0001	0	0.00001
G352V	0.00003	0	0	0	0	0.00007
D355N	0.00001	0	0	0	0	0.00002
H505R	0.00001	NA^*b*^	NA	NA	NA	NA
Y515C	0.000004	NA	NA	NA	NA	NA

a Minor allele frequencies (MAFs) of ACE2 SNVs among different populations were obtained from TOPMed, ExAC, or gnomAD.

b NA, information was not available in the databases.

## DISCUSSION

While ACE2 acts as a receptor for SARS CoVs, human ACE2 SNVs have been reported to be associated with cardiovascular diseases, hypertension, and diabetic mellitus, all of which are recognized as risk factors for severe clinical outcomes in COVID-19 patients (20–22). Genetic variations in the host ACE2 receptor may also influence the susceptibility or resistance to the SARS-CoVs. Several *in silico* studies suggested that ACE2 SNVs might affect the binding affinity to the S protein (15, 23, 24). However, only a few studies have reported on the biological importance of ACE2 SNVs in the cellular entry of SARS-CoVs (16, 25, 26), and the information on this topic is still limited. Previous studies demonstrated that some ACE2 SNV substitutions affected the binding affinity to RBD of the SARS-CoV-2 S protein (17, 18). In the present study, we investigated the effects of these SNV substitutions on actual viral entry into cells using pseudotyped VSIVs.

We found that the G352V substitution significantly enhanced the entry of SARS-CoV but affected that of SARS-CoV-2 only slightly. In contrast, the D355N substitution significantly reduced the entry of SARS-CoV but not SARS-CoV-2. Since the amino acid residue at position 352 in ACE2 is adjacent to a loop structure that is responsible for the interaction with the S proteins of SARS CoVs (27, 28), the G352V substitution likely affects the structure and/or molecular flexibility of this loop, which might result in enhanced binding to the S protein. Cryo-electron microscopy (Cryo-EM) of the structures of the ACE2 and S protein complexes revealed that the aspartic acid at position 355 of human ACE2 formed hydrogen bonds and van der Waals contacts with 3 (T486, T487, and G488) and 2 (T500 and G502) amino acid residues of SARS-CoV and SARS-CoV-2 S proteins, respectively (29). Differences in the number of van der Waals contacts might contribute to the differential effects of the D355N substitution between SARS-CoV and SARS-CoV-2. In another cocrystal structure of the SARS-CoV-2 S protein and ACE2, 22 residues in the ACE2 molecule are involved in the binding to the S protein of SARS-CoV-2 (29) and 6 of the 22 amino acid positions were reported to be SNV positions (S19P, T27A, E35D/K, E37K, M82I, and D355N). Another group demonstrated that the binding affinity to RBD of the SARS-CoV-2 S protein was increased in S19P, T27A, and E35D mutants and was reduced in E35K, E37K, M82I, and D355N mutants (30). However, these SNV substitutions, except E35K, did not significantly affect the viral infectivity in the present study (Fig. 2 and 4). Since a recombinant RBD molecule lacking all the other portions of the S protein was used in the previous binding assays (17, 30) one possible explanation is that structural and functional differences between the RBD molecule and the full-length whole S protein might affect the affinity or avidity to ACE2. Furthermore, Cryo-EM analyses revealed that the SARS-CoV-2 S protein had RBD-exposed conformation (open) and RBD-buried conformation (closed) and a high proportion of the S protein showed closed conformation (31, 32), which might weaken the effects of the substitutions in ACE2 when the whole S protein molecule is used for analyses. This difference between RBD and full-length S protein molecules could be one of the reasons for the lesser effects of the ACE2 substitutions on the SARS-CoV 2 entry than on the RBD binding capacity to ACE2.

The H505R substitution in ACE2 enhanced the entry of both SARS-CoV and SARS-CoV-2, including some VOCs. The Y515C substitution also tended to enhance the entry. These residues are not directly involved in the molecular surface that interacts with RBD of the S protein. The histidine residue at position 505 of ACE2 is thought to be a catalytic histidine based on a site-directed mutagenesis experiment for the ACE2 enzymatic activity (33). Furthermore, this amino acid residue may be important for the hydrogen bond to the tyrosine at position 515, which has been suggested to stabilize the carbonyl tetrahedral intermediate in the ACE2 molecule (33, 34). Thus, these SNV mutants might have altered enzymatic activities, while the structure of ACE2 might also be affected by the substitutions. However, a previous study reported that the ACE2 binding region of the SARS-CoV S protein was not interfered with inhibitors or substrates that induced large conformational changes in the receptor, suggesting that the enzymatic activity of ACE2 does not contribute to SARS-CoV S protein-mediated infection (35).

In the immunoblotting image (Fig. 2B), two bands were detected, which corresponded to unglycosylated (85- to 90-kDa) and glycosylated (110- to 120-kDa) forms of the ACE2 protein. N-glycosylation is required for proper ACE2 expression on the cell surface and efficient viral entry of SARS-CoV-2 (36). The ACE2 molecule has seven N-glycosylation sites (N53, N90, N103, N322, N432, N546, and N690) and a molecular dynamics simulation study reported that two glycosylation sites, N90 and N322, affected the binding capacity to the SARS-CoV-2 S protein (37). Another study showed that the mutation at T92 removing the N90-glycosylation motif enhanced the interaction with the S protein of SARS-CoV-2 (17). However, since the amino acid positions assessed in this study were not located in the reported N-glycosylation sites except for T92I and the majority of the detected bands were most likely glycosylated forms, N-glycosylation patterns might not affect the cell surface expression and binding efficiency of ACE2.

It was previously demonstrated that the H505R and Y515C ACE2 mutants showed weak binding affinity to RBD of the SARS-CoV-2 S protein (18). This discrepancy may also be due to the structural difference between RBD and full-length S protein molecules as discussed above. Alternatively, it may also be possible that weak binding capacity of ACE2 mutants does not necessarily cause reduced entry of the virus. A previous molecular dynamics analysis suggests that the ACE2 structural changes caused by some SNV substitutions likely affect the stability of the ACE2 molecule (38), which may influence the efficiency of membrane fusion. We speculate that H505R and Y515C mutants may have enhanced potential to support membrane fusion, which overwhelms their reduced binding to the S protein and results in increased entry of the virus.

Interestingly, the E35K substitution reduced the entry of SARS-CoV-2 variants with triple substitutions in their RBDs (i.e., Beta and Gamma) but not the other viruses tested, including the Alpha variant, which also had N501Y substitution, suggesting that the effect of the E35K substitution might be related to the K417N and/or E484K substitutions commonly found in Beta and Gamma variants. It has been shown that the lysine residue at position 417 forms a salt bridge with the aspartic acid at position 30 of ACE2, and the glutamic acid at position 484 interacts with the lysine at position 31 of ACE2 (Fig. 5B) (29, 39). The importance of the lysine at 417 and the glutamic acid at 484 for the interaction with ACE2 suggests the possibility that K417N and E484K substitutions negatively affect the binding to the ACE2 E35K mutant. Another study showed that the triple substitution (K417N, E484K, and N501Y) in the Beta variant changed the secondary structure and stability of RBD of the SARS-CoV-2 S protein (40). Taken together, the structural changes caused by these substitutions in RBD of the S protein might reduce the interaction with the ACE2 E35K mutant.

Genetic polymorphisms of the ACE2 and TMPRSS2 genes have been suggested to be involved in the disease outcomes of SARS-CoV-2 infection (10). Our findings support the notion that ACE2 SNVs may contribute to the enhancement or reduction of cell susceptibility to SARS CoVs, although the SNV substitutions that were found to be important in this study are rare in the current human population. It should also be noted that the effects of ACE2 SNVs cannot be determined solely from RBD binding assays, as demonstrated by the inconsistent results between previous RBD binding assays and viral entry assays in the present study. The limitation of our study is that we only focused on the single step of viral entry and the effects of ACE2 SNV substitutions were not very drastic; however, we assume that the differences among ACE2 variants may be enhanced in multiple replication cycles of authentic SARS-CoV and SARS-CoV-2. Furthermore, it still needs to be clarified whether ACE2 SNVs affect the clinical outcomes in COVID-19 patients. In the future, large-scale clinical studies of genetic variations are needed to confirm the significance of SNVs *in vivo* and to further understand possible relationships between host genetic factors and SARS-CoV-2 evolution.

## MATERIALS AND METHODS

### ACE2 SNV information.

Based on previous studies (17, 18), 29 SNV substitutions in ACE2 were selected for the analysis. The allele frequencies of ACE2 SNVs were extracted from TOPMed, ExAC, or gnomAD linked with the Single Nucleotide Polymorphism Database (dbSNP) on the National Center for Biotechnology Information (NCBI) website (https://www.ncbi.nlm.nih.gov/snp/, accessed on 1 December 2021). The NCBI ID numbers of these SNVs are as follows: rs73635825 (S19P), rs1244687367 (I21T), rs756231991 (E23K), rs1434130600 (A25T), rs4646116 (K26R), rs781255386 (T27A), rs778500138 (E35D), rs1348114695 (E35K), rs146676783 (E37K), rs1192192618 (Y50F), rs760159085 (N51D), rs1325542104 (M62V), rs1199100713 (N64K), rs755691167 (K68E), rs1256007252 (F72V), rs867318181 (E75G), rs766996587 (M82I), rs763395248 (T92I), rs1395878099 (Q102P), rs759579097 (G326E), rs143936283 (E329G), rs370610075 (G352V), rs961360700 (D355N), rs142984500 (H378R), rs751572714 (Q388L), rs762890235 (P389H), rs1016409802 (H505R), rs1352194082 (R514G), and rs1263424292 (Y515C).

### Cells.

Human hepatoma Huh-7 and human embryonic kidney HEK293T cells were cultured in Dulbecco modified Eagle medium (DMEM; Sigma-Aldrich) containing 10% fatal calf serum (FCS; Cell Culture Bioscience), 100 U/mL penicillin, and 0.1 mg/mL streptomycin (GIBCO) at 37°C in a 5% CO_2_ incubator.

### Plasmids.

Total RNA was extracted from Huh-7 cells with TRIzol (Invitrogen), and then a cDNA library was prepared using SuperScript IV (Invitrogen) and an ACE2 gene-specific primer (5′-CTAAAAGGAGGTCTGAACATCATCAGTGTT-3′). The coding region of the human ACE2 gene was amplified by PCR using the primers SacI-ACE2-F (5′-GCGGAGCTCGCCACCATGTCAAGC-3′) containing a SacI restriction site and NheI-ACE2-R (5′-CGCGCTAGCAAAGGAGGTCTGAAC-3′) containing an NheI restriction site. After sequence confirmation of the WT human ACE2 gene (DDBJ accession number NM_021804.3), this PCR product was inserted into a plasmid, pCAGGS, to express full-length ACE2 fused with a C-terminal 3×FLAG tag (pCAGGS-ACE2 3×FLAG). SNV mutants were produced by site-directed mutagenesis using KOD One polymerase (Toyobo) with primers containing the desired nucleotide substitutions. All mutations were confirmed by Sanger sequencing of the plasmids.

### VSIVs pseudotyped with S proteins of SARS CoVs.

Using a replication-incompetent VSIV containing the green fluorescent protein (GFP) instead of the receptor-binding VSIV glycoprotein (G) gene (VSVΔG*-G), VSIVs pseudotyped with S proteins of SARS CoVs (VSVΔG*-SCoV and VSVΔG*-SCoV-2) were generated as described previously (41, 42). Briefly, 24 h after transfection of HEK293T cells with pCAGGS expressing the S protein of SARS-CoV (Tor2 strain; GenBank accession number NC_004718.3) or SARS-CoV-2 (strain WHU01; GenBank accession number MN988668.1), the cells were incubated with VSVΔG*-G for 60 min at 37°C. After three washes with DMEM, the medium was replaced by DMEM with 10% FCS. After 24 h, the supernatants were harvested and stored at −80°C until use. Virus IUs in HEK293T cells were determined by counting the number of GFP-positive cells with an IN Cell Analyzer 2500HS (GE Healthcare). To produce VSIVs pseudotyped with the S proteins having the substitutions in RBD derived from the Alpha (N501Y), Beta (K417N, E484K, and N501Y), Gamma (K417T, E484K, and N501Y), and Delta (L452R and T478K) variants, the mutant S protein genes were constructed by site-directed mutagenesis with KOD One (Toyobo) based on the S protein gene of SARS-CoV-2 WHU01, which is an early isolate from Wuhan. Each mutation was confirmed by Sanger sequencing.

### Sodium dodecyl surface-polyacrylamide gel electrophoresis and Western blotting.

To check the expression levels of the ACE2 WT and SNV mutant proteins, HEK293T cells were transfected with expression plasmids encoding 3×FLAG-tagged ACE2 proteins or empty pCAGGS as a negative control. At 48 h posttransfection, these cells were washed with phosphate-buffered saline (PBS) three times and lysed with a radioimmunoprecipitation assay buffer (0.25 mM EDTA [pH 8.0], 25 mM Tris-HCl [pH 7.6], 150 mM NaCl, 1% NP-40, 1% sodium deoxycholate, 0.1% SDS). The supernatants were then collected after centrifugation. Each sample was mixed with 4× sample buffer (Bio-Rad) with 5% 2-mercaptoethanol, and solubilized proteins were separated by 10% SDS-PAGE. Separated proteins were then blotted on a polyvinylidene difluoride membrane (Merck Millipore). After blocking with 5% skim milk, the membrane was incubated with a mouse anti-FLAG M2 monoclonal antibody (Sigma-Aldrich, F1804) or mouse anti-β actin monoclonal antibody (AC15; Abcam) as a primary antibody for 1 h. After being washed with 0.05% Tween 20 in PBS (PBST), the membrane was incubated with horseradish peroxidase-conjugated goat anti-mouse IgG (Jackson ImmunoResearch, 115-035-062) as a secondary antibody for 1 h. After a washing step with PBST, the bound antibodies were visualized with Immobilon Western (Merck Millipore). Unglycosylated and glycosylated forms of the ACE2 protein were detected as approximately 85-kDa and 110- to 120-kDa bands consistent with a previous study, respectively (43, 44). The relative expression levels were analyzed using Amersham Imager 600 (GE Healthcare Japan).

### Virus entry assay.

HEK293T cells, which are known to lack expression of endogenous ACE2 (45), were seeded in 96-well plates (1.0 × 10^4^ cells per well) precoated with poly-l-lysine (Cultrex). After 24 h, the cells were transfected with 0.2 μg/well pCAGGS encoding FLAG-tagged ACE2 WT or SNV mutant proteins using TransIT-LT1 (Mirus). At 24 h posttransfection, these cells were infected with VSVΔG*-SCoV, VSVΔG*-SCoV-2, or VSVΔG*-G. Each virus was appropriately diluted to provide 300 to 400 IUs/well in HEK293T expressing WT ACE2. VSVΔG*-SCoV and VSVΔG*-SCoV-2 were treated with an anti-VSIV G monoclonal antibody [VSV-G(N)1-9] to abolish the background infectivity of parental VSVΔG*-G (46). After 24 h, the IUs were determined by counting the numbers of GFP-expressing cells by using an IN Cell Analyzer 2500HS (GE Healthcare). The relative infectivity was determined by setting the value of cells expressing ACE2 WT to 100%.

### Immunofluorescence assay.

HEK293T cells were seeded in a μ-Slide 8-Well Chamber Slide (iBidi GmbH) after precoating with poly-l-lysine (Cultrex). After 24 h, the cells were transfected with the pCAGGS encoding FLAG-tagged ACE2 proteins or empty pCAGGS. At 24 h posttransfection, the cells were washed with PBS and fixed with PBS containing 4% paraformaldehyde for 15 min. After a wash with PBS, the cells were incubated with PBS containing 3% bovine serum albumin for blocking for 1 h at room temperature. The cells were washed three times with PBST and then incubated with an anti-ACE2 recombinant rabbit monoclonal antibody (Invitrogen, SN0754) recognizing an epitope at amino acid positions 190 to 230 as the primary antibody for 1 h at room temperature. The cells were washed with PBST and then incubated with an Alexa Fluor 488-conjugated goat anti-rabbit IgG antibody (Molecular Probes) as a secondary antibody and counterstained with 1 μg/mL 4′,6-diamidino-2-phenylindole dihydrochloride (DAPI; Molecular Probes) for 1 h in the dark at room temperature. Images were acquired with a 63× oil lens objective on a Zeiss LSM700 inverted microscope using ZEN 2009 software (Carl Zeiss).

### Flow cytometry.

HEK293T cells were seeded in 6-well plates (2.0 × 10^4^ cells per well) precoated with poly-l-lysine (Cultrex). After 24 h, the cells were transfected with pCAGGS encoding FLAG-tagged ACE2 WT or SNV mutant proteins using TransIT-LT1. At 24 h posttransfection, these cells were washed with PBS and detached using 0.25% trypsin. Cells were fixed with PBS containing 4% paraformaldehyde for 15 min. After a wash with PBS, cells were incubated with an anti-ACE2 recombinant rabbit monoclonal antibody (Invitrogen, SN0754) for 1 h at room temperature. The cells were next stained with the Alexa Fluor 488-conjugated goat anti-rabbit IgG antibody (Molecular Probes) for 30 min at 4°C in the dark. After two washes, the surface expression of the exogenous ACE2 proteins was analyzed by using a FACSCanto flow cytometer (BD Biosciences) and FlowJo software (Tree Star).

### Statistical analysis.

All statistical analyses were performed using R software (version 3.6.0). To compare the relative infectivities, one-way analysis of variance followed by the Dunnett test was used. *P* values of <0.05 were considered statistically significant.
